# Implementation of an audit and feedback module targeting low-value clinical practices in a provincial trauma quality assurance program: a cost-effectiveness study

**DOI:** 10.1186/s12913-024-10969-2

**Published:** 2024-04-18

**Authors:** Blanchard Conombo, Jason R. Guertin, Jeffrey S. Hoch, Jeremy Grimshaw, Mélanie Bérubé, Christian Malo, Simon Berthelot, François Lauzier, Henry T. Stelfox, Alexis F. Turgeon, Patrick Archambault, Amina Belcaid, Lynne Moore

**Affiliations:** 1 Department of Social and Preventative Medicine, Université Laval, Québec City, Québec Canada; 2 Population Health and Optimal Health Practices Research Unit, Trauma – Emergency – Critical Care Medicine, Quebec University Hospital, Centre de Recherche du CHU de Québec-Université Laval, 18E Rue, Local H-012a, Québec City, Québec 1401G1J 1Z4 Canada; 3 Division of Health Policy and Management, Department of Public Health Sciences, University of California at Davis, Davis, CA USA; 4 Department of Medicine, University of Ottawa, Ottawa, ON Canada; 5 Faculty of Nursing, Université Laval, Québec City, Québec Canada; 6 Department of Family Medicine and Emergency Medicine, Université Laval, Québec City, Québec Canada; 7 Centre de Recherche Intégrée Pour Un Système Apprenant en Santé Et Services Sociaux, Centre Intégré de Santé Et de Services Sociaux de Chaudière-Appalaches, Lévis, Québec Canada; 8 Department of Anesthesiology and Critical Care Medicine, Division of Critical Care Medicine, Université Laval, Québec City, Québec Canada; 9 Department of Critical Care Medicine, Medicine and Community Health Sciences, O’Brien Institute for Public Health, University of Calgary, Calgary, AB Canada; 10 VITAM-Centre de Recherche en Santé Durable, Québec City, Québec Canada

**Keywords:** Audit and feedback, Cost-effectiveness analysis, Low-value care, Injury

## Abstract

**Background:**

Audit and Feedback (A&F) interventions based on quality indicators have been shown to lead to significant improvements in compliance with evidence-based care including de-adoption of low-value practices (LVPs). Our primary aim was to evaluate the cost-effectiveness of adding a hypothetical A&F module targeting LVPs for trauma admissions to an existing quality assurance intervention targeting high-value care and risk-adjusted outcomes. A secondary aim was to assess how certain A&F characteristics might influence its cost-effectiveness.

**Methods:**

We conducted a cost-effectiveness analysis using a probabilistic static decision analytic model in the Québec trauma care continuum. We considered the Québec Ministry of Health perspective. Our economic evaluation compared a hypothetical scenario in which the A&F module targeting LVPs is implemented in a Canadian provincial trauma quality assurance program to a status quo scenario in which the A&F module is not implemented. In scenarios analyses we assessed the impact of A&F characteristics on its cost-effectiveness. Results are presented in terms of incremental costs per LVP avoided.

**Results:**

Results suggest that the implementation of A&F module (Cost = $1,480,850; Number of LVPs = 6,005) is associated with higher costs and higher effectiveness compared to status quo (Cost = $1,124,661; Number of LVPs = 8,228). The A&F module would cost $160 per LVP avoided compared to status quo. The A&F module becomes more cost-effective with the addition of facilitation visits; more frequent evaluation; and when only high-volume trauma centers are considered.

**Conclusion:**

A&F module targeting LVPs is associated with higher costs and higher effectiveness than status quo and has the potential to be cost-effective if the decision-makers’ willingness-to-pay is at least $160 per LVP avoided. This likely represents an underestimate of true ICER due to underestimated costs or missed opportunity costs. Results suggest that virtual facilitation visits, frequent evaluation, and implementing the module in high-volume centers can improve cost-effectiveness.

**Supplementary Information:**

The online version contains supplementary material available at 10.1186/s12913-024-10969-2.

## Introduction

Low-value practices (LVPs) are tests and treatments that are not supported by evidence and may expose patients to physical and psychological harm [1, 2]. They have been estimated to consume up to 30% of healthcare resources in Canada [3] and in the US [4]. In 2013, an estimated $270 billion was wasted on excess healthcare services in the US [2]. From a patient and caregiver perspective, LVPs expose patients to physical and psychological harms, delays to effective treatment, and direct and indirect expenses [2, 5–8]. From a healthcare system perspective, they put strain on tight healthcare budgets and decrease the availability of scarce resources.

Recent literature suggests that interventions targeting the de-implementation of ineffective or harmful health interventions have the potential to reduce overuse and improve clinically important outcomes [9]. Among these are Audit and Feedback (A&F) interventions, defined as ‘a summary of clinical performance of healthcare over a specified period aimed at providing information to health professionals to allow them to assess and adjust their performance’ [10]. We now have extensive evidence of the effectiveness of A&F interventions, including those targeting de-implementation of LVPs. A systematic review including 140 randomized controlled trials (RCTs) estimated that A&F interventions resulted in close to 4.3% absolute increase in adherence to evidence-based care (IQR 0.5% to 16.0%) [11]. The effect of an A&F intervention appears to be larger when it targets de-implementation of low-value practices (absolute decrease of 10.5%). This review also revealed that A&F effectiveness is influenced by its design and delivery [11]. The World Health Organisation recently expressed concern about the major knowledge gap on the cost and cost-effectiveness of A&F interventions [12], and recommended that implementation of these interventions be informed by data on their cost-effectiveness [13]. Despite this, most A&F interventions, including those used across Canadian trauma systems, are implemented without evidence on their cost-effectiveness [12, 14]. A 2022 systematic review summarized evidence on the economic value of A&F interventions in healthcare [15] and found that they have a high potential to be cost-effective. However, authors only identified economic evaluations for 6% of A&F trials, methodological quality of these evaluations was low, and authors concluded that model-based simulations were urgently needed to assess the impact of A&F characteristics on cost-effectiveness to inform optimal A&F design.

Trauma systems are a favorable setting for de-implementation interventions as they possess many documented facilitators including quality improvement teams with medical leadership, routinely-collected clinical data, and performance linked to accreditation [16]. Furthermore, potential gains are huge due to the resource-intensive nature of trauma care. Trauma systems are thus the ideal setting to advance knowledge on de-implementation. Our research team recently published a list of quality indicators targeting LVPs in acute trauma care [17, 18]. We aim to evaluate the cost-effectiveness of an A&F module targeting the de-implementation of these LVPs in an integrated Canadian trauma system and to assess the impact of A&F characteristics on cost-effectiveness.

## Methods

We conducted an economic evaluation according to the Canadian guidelines for the Economic Evaluation of Health Technologies [19], and results are reported following the CHEERS 2022 statement [20]. The study protocol was developed with a project advisory committee including two emergency physicians (CM, EM), two trauma surgeons (TR, NY), three critical care physicians (FL, AFT, HTS), a neurosurgeon (PLB), a spine surgeon (JP), an orthopedic surgeon (ML), two trauma service managers (MB, CR), a trauma registry co-ordinator (AB), and epidemiologist (LM), and two health economists (JRG, JSH). The protocol was approved a priori by all co-authors, members of the advisory committee, a granting agency peer-review committee (Canadian Institutes of Health Research project #353374) and the CHU-de Québec – Université Laval research ethics committee.

### Setting

Our economic evaluation is based on a hypothetical A&F module embedded in the *Québec Trauma Care Continuum*, a provincial regionalized trauma system comprising 57 adult trauma centers of which 3 are level I (highly specialized urban centers), 5 are level II (similar capacity to level I but in smaller cities), 21 are level III (hospitals in small towns transferring most major trauma to level I/II centers after stabilization), and 28 are level IV (rural community hospitals). All centers undergo mandatory, periodic verifications in line with designation, conducted by the provincial healthcare quality agency, *Institut national d’excellence en santé et services sociaux* (*INESSS*) and overseen by the Ministry of Health and Social Services [21]. Verification includes A&F on adherence to *high-value* care and risk-adjusted outcomes. Local trauma committees in each center are required to ensure the quality of the trauma program according to designation requirements. Committees include the program medical director (Chair), the program manager, heads of critical care, emergency and surgical departments, heads of multidisciplinary services, and a hospital administrator. Quality improvement activities include trimestral committee meetings with chart review, development of local care protocols, and discussions with clinical and administrative leads locally and at referring centers to identify improvement strategies. Formal letters of agreement are signed by heads of clinical departments to operate changes in their services when required.

### Intervention and comparator

We compared a hypothetical scenario in which an A&F module targeting LVPs is implemented in the Québec trauma system to a status quo scenario in which the A&F module is not implemented.

#### Comparator (status quo scenario)

The study comparator is the A&F intervention currently in place in the Québec Trauma Care Continuum, designed by the provincial healthcare quality agency using the US Agency for Healthcare Research and Quality guidelines [22]. This A&F intervention targets trauma committees in each trauma center and, as explained above, currently includes modules for adherence to *high-value* practices (15 quality indicators) and optimal outcomes (3 quality indicators). The A&F intervention currently in place consists of:

- Quality reports disseminated via a Web platform to local trauma committees and hospital boards of directors produced using trauma registry data.
- Web links to user-friendly information sheets including definitions for quality indicators and references supporting each indicator.
- Information sheets and Web capsules with guidelines on how the results should be interpreted and acted upon.
- A case revision tool integrated into the trauma registry.

Within 6 months of reception of the report, committees are required to submit an action plan proposing improvement strategy for quality indicators for which they are identified as negative outliers.

#### Intervention

The study intervention is an A&F module targeting LVPs (6 quality indicators) (http://www.ohri.ca/auditfeedback/laboratories/). The 6 quality indicators were selected using the results of an expert consensus study [23] and an indicator validation study using data from the Quebec trauma registy [24].

In the base case scenario, the module includes the components already in place described in the status quo scenario, applied to quality indicators on LVPs. We attributed a 5-year lifespan to the A&F module as current literature recommends that quality indicators be updated every five years [25]. To account for the 5-year lifespan of the A&F module and its potential benefits one year beyond its lifespan, we used a 6-year time horizon.

### Type of economic evaluation

**Fig. 1:**
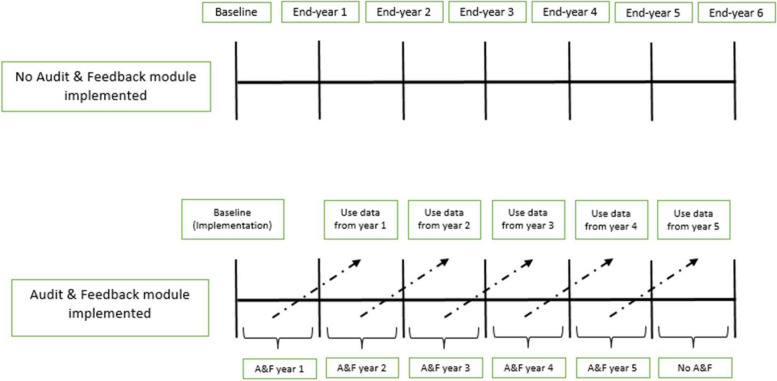
Decision-analytic model. In the status quo scenario, there is no implementation of the A&F module targeting LVPs. In the intervention scenario, an A&F module targeting LVPs is implemented at baseline (at the beginning of the 1st year). “Use data from year 1” means that data on the effectiveness and costs of A&F module from the 1st year are available at the beginning of the second year and so on for the following years

For this early economic evaluation, i.e., an evaluation prior to the implementation of the module, a probabilistic static decision analytic model was developed to estimate the incremental cost-effectiveness ratio (ICER) of the A&F module compared with status quo scenario in which the A&F module is not implemented for patients with acute injury (Fig. 1). We considered the Québec Ministry of Health perspective.

### Effectiveness

The incremental effectiveness of the A&F module was estimated as the incremental number of LVPs avoided. Plausible ranges of percent reductions in LVPs were obtained from the 2012 Cochrane review, which presented effectiveness of A&F interventions as medians and interquartile ranges [26]. Specifically, we used the pooled estimate of effectiveness specific to deimplementation interventions based on 29 studies. For the purposes of our analysis, these values were used to estimate mean effectiveness and associated standard errors using a method based on highly-cited recommendations [27, 28].

### Costs

The incremental costs of the A&F module over the comparator were estimated by summing the implementation costs of the A&F module over its 5-year lifespan and the potential reduction in resource utilization for all LVPs, valued in costs and estimated between years 2 and 6. The implementation costs were determined by identifying all non-recurrent and recurrent costs related to the implementation of the A&F module including data validation and analyses, report production and validation, administrative costs, and follow-up in local trauma committees (Table 1). The potential reduction in resource utilization was estimated by multiplying the hypothesized reduction in the frequency of the LVPs by their average costs. Detailed information on how practices were costed are available elsewhere [29]. Briefly, we estimated direct healthcare costs for each LVP from the Ministry of Health perspective using an activity-based costing approach. Activity-based costing involves multiplying unit costs of specific activity centres by the corresponding units of resources used. This method provides an estimate of hospital resource use by activity center, consistent with Grading of Recommendations Assessment, Development and Evaluations guidelines [30–32]. All costs are expressed in 2020 Canadian dollars. We report our study following the Consolidated Health Economic Evaluation Reporting Standards statement [29].

**Table 1:** Resources used and costs associated with status quo scenario for a level I trauma center (A&F targeting 15 *high-value* practices and 3 outcomes) and additional costs of A&F module (targeting 6 *LVPs*) per A&F cycle (one year)^a^

**Role**	**Description**	**Estimated costs ($CAN 2020)**
**Costs associated with status quo scenario**		
**Report preparation (Costs for all trauma centers)**		
Biostatistician	- Data manipulation, validation and analysis, and preparing graphs (Biostatistician: 2 days per week for 6 months; Research coordinator: 3 days per week for 6 months)	$33,060
Research coordinator		
Research supervisor	- Validating materials for INESSS (6 h)	
**Act on the results (Costs for a level I trauma center)**		
Medical coder	- Prepare patient charts for review (20 h for 18 quality indicators)	$10,555
Trauma program manager	- Three days of data analysis (7 h per day per person involved)	
Two trauma program medical directors		
**Local trauma committee meeting (Costs for a level I trauma center)**		
Trauma program manager	- Patient chart revision; - Discuss problems and potential solutions with healthcare providers involved (to identify improvement strategies); (2 h per meeting for 5 meetings in one evaluation cycle)	$7,445
Hospital administrator		
Three physicians		
Medical coder		
Laboratory technician or multidisciplinary services		
**Report production for INESSS—Action plan (Costs for a level I trauma center)**		
Trauma program manager	- Two weeks of full-time work by the trauma service (7 h per day) coordinator to produce the action plan	$10,561
Medical directors	- Report validation by heads of 10 medical services (3 h per person)	
**Total costs for a Level I center with 18 indicators in the status quo scenario**		**$28,562**
**Additional costs associated with implementation of A&F module targeting 6 LVPs**		
Costs of adding 6 quality indicators for a level I trauma center		$9,521
Costs of adding 6 quality indicators to all three level I centers^b^		$28,562
Additional costs for report preparation for 6 quality indicators		$11,020
Total costs of adding 6 quality indicators to all 57 adult’s trauma centers (plus report preparation)		$135,363

*LVP* Low-value practices, *A&F* Audit and Feedback, *INESSS* Institut national d’excellence en santé et services sociaux

^a^Estimated using expert consultation

^b^The additional costs associated with the A&F module targeting 6 LVPs for trauma centers level II, III and IV were calculated as a proportion of the average annual number of admissions compared to level I trauma centers

### Incremental cost-effectiveness ratio

The incremental cost-effectiveness ratio (ICER) was estimated by dividing the incremental costs (or savings) of the A&F module by its incremental effectiveness. Results are reported as the incremental cost per LVP avoided.

### Discount rate

All future costs and benefits were discounted at a rate of 1.5% as recommended by current Canadian guidelines [19].

### Scenario analyses

Our advisory committee identified 6 scenario-specific sensitivity analyses based on published evidence of A&F effectiveness and context-specific considerations (#1, 2, 3, 6) [24, 26] as well as Canadian guidelines on economic evaluation (#4, 5):

1. Adding a virtual facilitation visit once per cycle to help trauma committees identify barriers and facilitators and use them to identify improvement strategies for their action plan; [15, 26]
2. Increasing feedback frequency from annually to monthly, as assessed in the systematic review; [26]
3. Implementing the module only in high-volume trauma centers (i.e., level I and II);
4. Varying the discount rate between 0 and 5%, as recommended by current Canadian guidelines; [19]
5. Increasing the lifespan of the A&F module to 10 years (Supplementary Fig. 1) to take into account the effect of time on module effectiveness.
6. Increasing the costs of LVPs by 100% to account for lack of complete data on physician billing and unit costs that underestimate market prices. This is based on evidence that physician billing represents approximately 56% of hospital costs in Canada (https://www.cihi.ca/sites/default/files/document/nhex-trends-2020-narrative-report-en.pdf).

### Analyses

We present the ICER based on the results of probabilistic sensitivity analysis (PSA) as recommended in the Canadian Guidelines for the Economic Evaluation of Health Technologies [19]. In the PSA, model parameters were represented by distributions of possible values rather than point estimates to address parameter uncertainty. All parameters and their distributions are presented in Table 2. Parameter distributions were randomly sampled 10,000 times. Results were summarized using cost-effectiveness acceptability curves (CEACs) and the cost-effectiveness acceptability frontier (CEAF) [33]. We used Excel software (Microsoft Office 2019 Professional Plus) to construct a decision model, to analyze base case results, and conduct PSA.

**Table 2:** Key parameters and their distributions

**Parameters**	**Base case value**	**SD**	**Distribution**^**a**^	**Source of information**
**Mean cost of LVPs (2020 CAN dollars)**				
Head CT in low-risk patients	89	---	---	AS471 (https://www.donneesquebec.ca/recherche/dataset/as-471-rapports-financiers-annuels-des-etablissements), RAMQ (https://www.ramq.gouv.qc.ca/fr/professionnels/medecins-specialistes/manuels/Pages/remuneration-acte.aspx)
Cervical spine CT in low-risk patients	123	---	---	AS471 (https://www.donneesquebec.ca/recherche/dataset/as-471-rapports-financiers-annuels-des-etablissements), RAMQ (https://www.ramq.gouv.qc.ca/fr/professionnels/medecins-specialistes/manuels/Pages/remuneration-acte.aspx)
Whole body CT in minor or single-system injury	296	---	---	AS471 (https://www.donneesquebec.ca/recherche/dataset/as-471-rapports-financiers-annuels-des-etablissements), RAMQ (https://www.ramq.gouv.qc.ca/fr/professionnels/medecins-specialistes/manuels/Pages/remuneration-acte.aspx)
Post-transfer repeat CT	98	---	---	AS471 (https://www.donneesquebec.ca/recherche/dataset/as-471-rapports-financiers-annuels-des-etablissements), RAMQ (https://www.ramq.gouv.qc.ca/fr/professionnels/medecins-specialistes/manuels/Pages/remuneration-acte.aspx)
Neurosurgical consultation for mild complicated TBI	113	---	–-	AS471 (https://www.donneesquebec.ca/recherche/dataset/as-471-rapports-financiers-annuels-des-etablissements), RAMQ (https://www.ramq.gouv.qc.ca/fr/professionnels/medecins-specialistes/manuels/Pages/remuneration-acte.aspx)
Spine service consultation for isolated thoracolumbar transverse process fractures	115	---	---	AS471 (https://www.donneesquebec.ca/recherche/dataset/as-471-rapports-financiers-annuels-des-etablissements), RAMQ (https://www.ramq.gouv.qc.ca/fr/professionnels/medecins-specialistes/manuels/Pages/remuneration-acte.aspx)
**Costs of A&F module (2020 CAN dollars)**				
Yearly cost of the A&F module	135,363	---	---	Expert consultation (Table 1)
**Mean annual number of LVPs**				
Head CT in low-risk patients	458	53.9	Normal	Moore et al. [24]
Cervical spine CT in low-risk patients	215	39.6	Normal	Moore et al. [24]
Whole body CT in minor or single-system injury	295	46.3	Normal	Moore et al. [24]
Post-transfer repeat CT	236	34.6	Normal	Moore et al. [24]
Neurosurgical consultation for mild complicated TBI	286	39.0	Normal	Moore et al. [24]
Spine service consultation for isolated thoracolumbar transverse process fractures	16	10.0	Normal	Moore et al. [24]
**Parameters regarding the effectiveness of the A&F module**^**b**^				
• Year 1	13.9%	10%	Beta	Ivers et al. [26]
• Year 2	13.9%	10%		
• Year 3	13.9%	10%		
• Year 4	13.9%	10%		
• Year 5	13.9%	10%		
**Other parameters**				
Discount rate	1.5%	---	---	CADTH [19]
Time horizon	6 years	---	---	Expert consultation

*AS471* hospital financial reports, *CADTH* Canadian Agency for Drugs & Technologies in Health, *LVP* Low-value practice, *RAMQ* Régie de l’assurance maladie du Québec, *SD* Standard Deviation

^a^To be used in the probabilistic sensitivity analyses

^b^The A&F module would reduce the volume of LVPs by around 13.9% each year over its 5-year lifetime

## Results

**Fig. 2:**
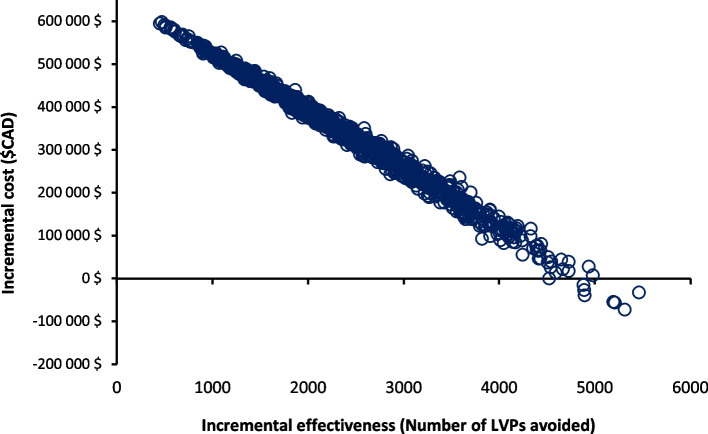
Probabilistic sensitivity analysis comparing A&F module and status quo scenario (no A&F module targeting LVPs). The x-axis represents the incremental effectiveness, number of LVPs avoided. The y-axis represents the incremental costs between A&F module and status quo scenario. Each circle represents a single simulation for a total of 10,000 simulations

**Fig. 3:**
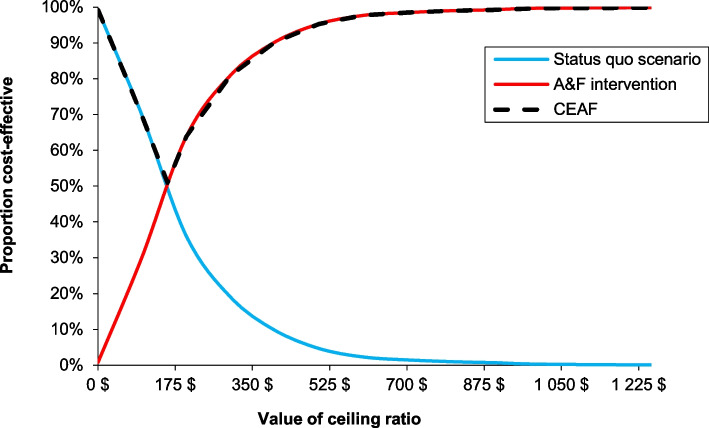
Cost-effectiveness acceptability curve (CEAC) between A&F module and status quo scenario and cost-effectiveness acceptability frontier (CEAF). The x-axis represents the willingness-to-pay (WTP) for each LVP avoided. The y-axis represents the percentage of simulations in which the A&F module is cost-effectiveness relative to the status quo scenario at different WTP threshold. The switch point where the A&F module became a cost-effective intervention corresponds to $160 per LVP avoided, equal to the ICER estimate. A&F module became 100% cost-effective at a WTP of $1000 per LVP avoided. The A&F module had the highest expected net benefit, for all values of WTP greater than the ICER. At our ICER estimate, 50% of the distribution of ICERs were cost-effective

The mean costs of the A&F module and status quo scenario were $1,480,850 and $1,124,661 respectively. The associated average number of LVPs were 6,005 for the A&F module and 8,228 for status quo scenario. The implementation of the A&F module is associated with a reduction of approximately 2,223 LVPs. The ICER for the A&F module versus status quo scenario was $160 per LVP avoided (Table 3). The results of the PSA plotted on a cost-effectiveness plane (Fig. 2) show that most of the points in the scatter plot are located in the Northeast quadrant, indicating that the A&F module has a potential to be cost-effective given a decision maker’s willingness-to-pay (WTP). The cost-effectiveness acceptability frontier indicates that A&F module is cost-effective in 50% of our iterations at a WTP of $160 per LVP avoided (Fig. 3).

**Table 3:** Results of economic evaluation

**Interventions**	**Expected cost**	**Incremental cost**	**Expected number of LVPs**	**Incremental** **Number of LVPs**	**ICER**
A&F module	$1,480,850	$356,189	6,005	-2,223	$160 per LVP avoided
Status quo scenario	$1,124,661	-	8,228	-	-

All costs are expressed in 2020 CAN$

### Scenario analyses

Adding a virtual facilitation visit to the A&F module (one visit per A&F cycle) would reduce the estimated ICER (improve its cost-effectiveness profile compared to the base case scenario) to $108 per LVP avoided (Table 4). More frequent feedback (monthly) is associated with a slight improvement in its cost-effectiveness profile ($154 per LVP avoided). The A&F module is more cost-effective ($48 per LVP avoided) when only high-volume trauma centers are considered for the implementation of the module. Similarly, an increase in the costs of LVPs by 100% and a longer time horizon would lead to a reduction in the ICER to $10 and $106 per LVP avoided, respectively. On the other hand, a discount rate of 5% increases the ICER to $199 per LVP avoided (Table 4).

**Table 4:** Scenario analysis

**Scenarios**	**Costs difference (2020 CAN $)**	**LVPs avoided**	**ICER (Costs per LVP avoided)**
A&F characteristics			
Adding virtual facilitation visits^a^	$301,849	-2,799	-$108
Increasing frequency of feedback to monthly^b^	$666,709	-4,333	-$154
Implementing in high-volume trauma centers only (level I and II)^c^	$79,298	-1,646	-$48
Economic evaluation assumptions			
Discount rate of 0%	$307,662	-2,625	-$117
Discount rate of 5%	$382,858	-1,927	-$199
10 year-lifespan	$571,575	-5,399	-$106
Increasing the costs of LVPs by 100%	$23,385	-2,371	-$10

^a^The addition of virtual facilitation visits would increase the cost of the intervention by $3,804 per year (salaries of two professionals for a one-hour visit in each center) with an efficiency that increases by 1.51 percentage points each year compared to the base-case scenario [26]

^b^A monthly feedback frequency would increase the module's effectiveness by 9.65 percentage points each year [26] compared with the base-case scenario, and increase annual costs by $121,220 (cost of producing 11 additional reports per year per center)

^c^Intervention costs for level III and IV trauma centers are excluded ($353 270)

## Discussion

The results of this early economic evaluation suggest that the addition of an A&F module targeting LVPs to a provincial trauma quality assurance program over a time horizon of 6 years is associated with an ICER of $160 per LVP avoided. In analyses that simultaneously accounted for uncertainty in all key model parameters, 50% of simulations were cost-effective at a WTP of $160 per LVP avoided. The A&F module is more cost-effective with the addition of facilitation visits, frequent evaluation and if restricted to high-volume trauma centers.

Our study fills a major knowledge gap on the potential cost-effectiveness of A&F interventions to de-implement low-value care. Comparison of our results with the literature on acute trauma care is difficult, because there are no studies that have assessed the cost-effectiveness profile of A&F interventions in the context of acute injury care. However, a 2022 systematic review on the economic value of A&F interventions in various health areas summarized results of 35 studies that compared different A&F strategies targeting health professionals compliance with desire practices or patient health outcomes [15]. The results of this systematic review mirror our findings. Of 14 cost-effectiveness analyses based on changes in compliance to desired practice from the public healthcare payer perspective, 12 (86%) studies found that the A&F interventions were more costly but more effective than the comparator [15]. From studies assessing de-implementation of LVPs [34, 35], A&F interventions were associated with a reduction in the overuse of LVPs and had the potential to be cost-effective [34, 35]. Four (28%) studies included in the review conducted simulations to assess the influence of A&F characteristics on cost-effectiveness in scenario analyses [34–37]. Despite having different comparator groups (do-nothing scenario), these studies also observed improved cost-effectiveness when facilitation visits are added to A&F intervention [35] and the time horizon of the intervention is increased to 9 months (4 to 9 months) [34]. In addition, our study provides evidence that cost-effectiveness of A&F interventions may be improved by increasing frequency and restricting the intervention to high volume hospitals.

### Strengths and limitations

Our study is based on effectiveness parameters from a meta-analysis on over 140 RCTs on the effectiveness of A&F interventions in different healthcare settings [11], on observed data on the frequency of LVPs [24], and on costs based on a mature, province-wide quality assurance program (https://www.donneesquebec.ca/recherche/dataset/as-471-rapports-financiers-annuels-des-etablissements). In addition, we conducted extensive sensitivity analyses and a range of scenario analyses to evaluate the robustness of our results and to assess the influence of key A&F characteristics on its cost-effectiveness profile. Despite these strengths, our results should be interpreted within the context of the study’s limitations. First, our evaluation is based on estimates of effectiveness from a meta-analysis published in 2012. While this study represents the most up-to-date evidence synthesis available (the Cochrane review is yet to be updated [38] and systematic reviews published more recently have not included meta-analyses) [39], it does not include the most recent evidence. Furthermore, while we used estimates specific to deimplementation interventions from the review, none of the studies were specific to trauma, none evaluated an intervention delivered in the context of accreditation, and none compared a deimplementation module in a system with an A&F intervention already in place. Furthermore, risk of bias was low for only 31% of included studies. The estimate used may therefore represent an underestimate or overestimate of the true effectiveness. Second, we conducted an early economic evaluation to assess if a hypothetical A&F module could be cost-effective and, if so, under which conditions. As such, the results of our economic evaluation provide encouragement that the true ICER of the intervention were it to be designed and implemented in the Québec trauma system might be promising as well. We used a broad range of scenarios and parameter values within our probabilistic sensitivity analyses but attributed the same weight to all scenarios analyzed. The base-case scenario will not necessarily be the one that will be implemented. However, intervention costs were based on resources currently used in the Québec trauma care continuum and opportunity costs related to LVPs avoided were based on observed baseline frequencies. We plan to conduct an economic evaluation after our cluster randomized trial (funded and currently underway) to assess the true (observed) cost-effectiveness of the intervention in a pragmatic setting. Third, opportunity costs of LVPs avoided did not fully account for physician fees and were based on unit costs that are known to underestimate their true costs. In addition, we did not account for potential resource repercussions of LVPs, for example, re-imaging due to uncertain findings or treatment of clinically nonsignificant incidental findings. Our scenario analysis where costs associated with LVPs were increased by 100% probably better reflects the Québec Ministry of Health perspective; the large decrease in the ICER ($160 to $10) suggest that opportunity costs related to LVPs are an important determinant of the cost-effectiveness of an A&F module targeting de-implementation. Furthermore, we only considered direct healthcare costs associated with the two competing strategies and did not factor in the effects of indirect costs (e.g., time off work for patients) from LVPs, which would also have led to an underestimation of the intervention’s cost-effectiveness. Fourth, our study is based on the single healthcare payer model, and it is uncertain if our findings would be applicable to other jurisdictions with alternate payer systems. Also, physicians in Canada receive payments based on fee for service that is periodically negotiated [40], so our results are dependent on current unit costs in our system and may not apply well in non-universal health systems or other jurisdictions with different structures. Fifth, in the absence of evidence indicating otherwise, our base case scenario was based on the strong assumption that effectiveness was the same for all 6 indicators. However, in probabilistic sensitivity analyses, we allowed the effectiveness of LVP to vary independently. Sixth, we were unable to take account of the uncertainty of the cost estimates of implementing the A&F module, derived from expert consultation, which we anticipate may have been underestimated. Finally, we deliberately focused on adherence to desired practice (LVPs avoided) rather than health outcomes (e.g., adverse events) due to lack of available data associated with utility/disutility of LVPs for trauma patients. Nevertheless, a strong argument can be made for focus on the measurement of LVPs avoided for assessment of the quality of our A&F intervention, as they relate most closely to actions that are within the control of healthcare professionals. Indeed, economic evaluations of similar A&F interventions have obtained more meaningful results with similar intermediate outcomes than with Quality-Adjusted Life Years (QALYs) [41]. Studies have also demonstrated that reducing LVPs will reduce physical harms and adverse events [42–44]. However, this probably led to an underestimation of the true cost-effectiveness profile of our A&F module as health outcomes or negative health consequences of LVPs are not considered in the measure of effectiveness [15].

### Potential impact

The outcome parameter used in decision model (LVPs avoided) is unique and does not have an explicit cost-effectiveness threshold associated with it. Therefore, the decision to invest in the intervention will be based on the decision-makers willingness-to-pay, i.e., would they be prepared to invest 160$ per LVP avoided? However, the decision should also be based on other considerations, e.g., opportunity costs are likely to be greater than those estimated, cost-effectiveness may be increased if virtual facilitation visits are added, if the frequency of evaluations are increased, and if the intervention is restricted to high-volume trauma centers (level I and II). The intervention has the potential to lead to a global awareness of healthcare overuse and therefore a decrease in other LVPs [24].

## Conclusion

Our economic evaluation suggests that an A&F module targeting de-implementation, integrated into a provincial quality-assurance program, has a high potential to reduce LVPs while increasing total healthcare costs, with an ICER of $160 per LVP avoided. Results suggest that virtual facilitation visits, frequent evaluation and implementing the intervention only in high-volume centers increase cost-effectiveness. However, its economic potential is likely underestimated in this study due to opportunity costs that were underestimated (costs of LVPs) or not accounted for (indirect costs, health outcomes, and long-term consequences). The findings of the present study may inform the development of A&F interventions targeting de-implementation and they demonstrate the feasibility of conducting early economic evaluations to inform optimal A&F intervention design.

### Supplementary Information

**Supplementary Material 1. **

## Data Availability

Quebec Trauma Registry is subject to a third-party restriction (Quebec Ministry of Health and Social Services).

## Abbreviations

LVPs: Low-value practices
ICER: Incremental cost-effectiveness ratio
A&F: Audit and Feedback
RCT: Randomized Controlled Trial
WTP: Willingness-to-pay
CT: Computed tomography
IQR: Interquartile Range
CEAC: Cost-effectiveness acceptability curve
CEAF: Cost-effectiveness acceptability frontier
PSA: Probability sensitivity analysis
