# Supplementary material for: Implementation of an audit and feedback module targeting low-value clinical practices in a provincial trauma quality assurance program: a cost-effectiveness study
Source: BMC Health Serv Res. 2024 Apr 18;24:479. doi: 10.1186/s12913-024-10969-2 (PMC11025277; doi:10.1186/s12913-024-10969-2)
Supplement: Supplementary file 1 — Supplementary Material 1. [file 12913_2024_10969_MOESM1_ESM.docx]

**Supplementary File**


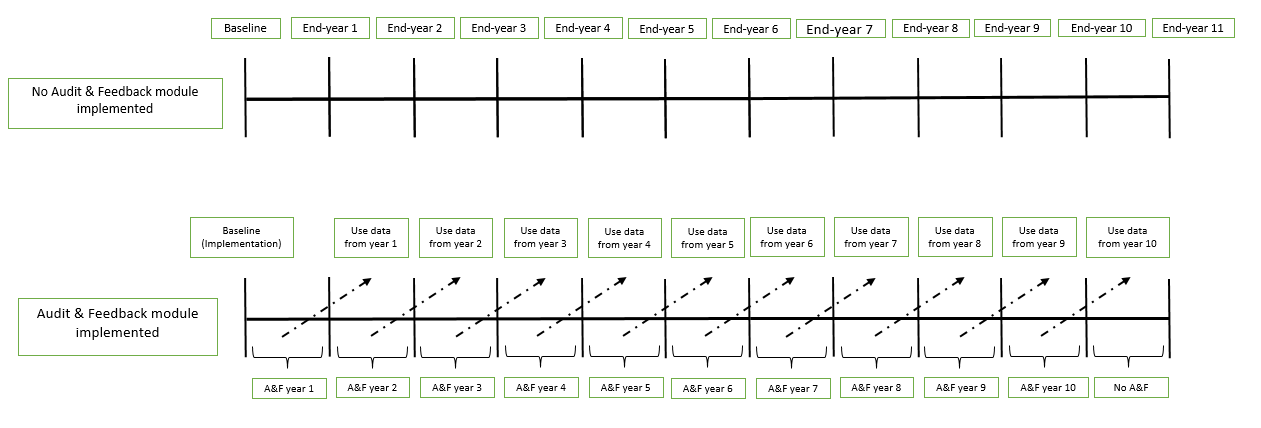


**Supplementary Figure 1.** A&F module over 10-year lifespan

**Supplemental Table 1.** Selected low-value clinical practices and their definitions^[[1]](#footnote-1)^

| **Selected low-value clinical practices** | **Definition** |
| --- | --- |
| Head CT in adults with mild TBI with no indication on a validated clinical decision rule | Adults at low risk on the Canadian CT Head Rule who had a head CT in the ED |
| Cervical spine CT in patients with no indication on a validated clinical decision rule | Adults at low risk on the Canadian CT Spine Rule who had a cervical spine CT in the ED |
| Post-transfer repeat CT in trauma patients with no disease progression and no additional details needed | Transferred adults with a CT in a referral center who received a CT in the same body region in the ED of the receiving center despite having no disease progression |
| Neurosurgical consultation in adults with acute mild complicated TBI not on anticoagulation therapy | Adults admitted with a diagnosis of mild complicated TBI (no clinically significant intracranial lesions) who received a neurosurgical consultation |
| Spine service consultation for isolated L1-L4 transverse process fractures | Adults with an acute isolated thoracolumbar transverse process fracture who received a spine service consultation |
| Whole body CT in adults with minor or single-system trauma | Hemodynamically stable adults with GCS≥13, no high-energy mechanism, and either minor trauma (ISS > 12) or single system trauma (not more than one injured body region with AIS≥3) with a whole-body CT in ED |

CT, computed tomography; ED, emergency department; TBI, traumatic brain injury; ISS, injury severity score; AIS, Abbreviated Injury Scale score; GCS, Glasgow Coma Scale

1. Moore L, Berube M, Tardif P, et al. Quality indicators targeting low-value clinical practices in trauma care: an expert consensus study. *Jama Surgery (in press).* 2022. [↑](#footnote-ref-1)
